# Phase I dose-escalation study of the HSP90 inhibitor AUY922 in Japanese patients with advanced solid tumors

**DOI:** 10.1007/s00280-014-2521-x

**Published:** 2014-07-25

**Authors:** Toshihiko Doi, Yusuke Onozawa, Nozomu Fuse, Takayuki Yoshino, Kentaro Yamazaki, Junichiro Watanabe, Mikhail Akimov, Matthew Robson, Narikazu Boku, Atsushi Ohtsu

**Affiliations:** 1 National Cancer Center Hospital East, 6-5-1 Kashiwanoha, Kashiwa, Chiba 277-8577 Japan; 2 Shizuoka Cancer Center, 1007 Shimonagakubo, Nagaizumi-cho, Sunto-gun, Shizuoka, 411-8777 Japan; 3 Novartis Pharma AG, 4002 Novartis Campus, Basel, Switzerland; 4 Novartis Pharma K.K., 4-12-24, Nishi-azabu, Minato-ku, Tokyo, 106-0031 Japan

**Keywords:** AUY922, Clinical trial, HSP90, Japanese, Phase I

## Abstract

**Purpose:**

AUY922 is a potent non-geldanamycin inhibitor of heat-shock protein 90. This study was carried out in Japanese patients to determine the maximum tolerated dose (MTD), and to characterize safety, tolerability and pharmacokinetics of single-agent AUY922.

**Methods:**

Japanese patients with advanced solid tumors whose disease had progressed on at least one line of standard therapy, or for whom no standard therapy existed, were treated with AUY922 (intravenous, once-weekly, 28-day cycle, starting dose 8 mg/m^2^).

**Results:**

Thirty-one patients were treated. Two DLTs were reported in one patient of the 54 mg/m^2^ cohort; fatigue and decreased appetite (both Grade 3, resolving to Grade 1 within 8 days). No MTD was determined, and the dose recommended for Phase II studies was determined to be 70 mg/m^2^ once-weekly. Most common drug-related toxicities were diarrhea, night blindness and nausea. Grade 1 and 2 visual toxicities at high AUY922 doses  ≥22 mg/m^2^ were observed. Ten patients (32 %) achieved a best overall response of stable disease, and one patient (3 %) achieved a confirmed partial response.

**Conclusion:**

Overall, AUY922 exhibited acceptable toxicities and demonstrated potential clinical activity in Japanese patients, with similar safety and pharmacokinetic profiles to those reported in a preceding global Phase I study in Western patients (CAUY922A2101).

## Introduction

Heat-shock proteins (HSPs) are molecular chaperones that assist in the structural formation, folding and activation of a wide variety of oncogenic client proteins involved in diverse cellular processes such as apoptosis, proliferation, signal transduction and transcription control [1–4]. These client proteins include human epidermal growth factor receptor 2 (HER2), estrogen receptor, epidermal growth factor receptor, platelet-derived growth factor receptor, vascular endothelial growth factor, AKT, c-KIT and c-MET [1, 2]. HSP90 is the most abundant molecular chaperone and is essential for cell survival, proliferation and apoptosis. These processes are significantly affected by HSP90 inhibition, and therefore, HSP90 inhibitors are considered to have a strong therapeutic potential in a wide variety of tumor types [5]. Indeed, HSP90 inhibitors degrade a variety of oncogenic client proteins [6–8]. In addition, HSP90 inhibitors show synergy with various chemotherapeutic agents in murine tumor models and sensitize tumor cells to their cytotoxic effects [6–8].

AUY922 (5-[2,4-dihydroxy-5-isopropyl-phenyl]-*N*-ethyl-4-[4-(morpholinomethyl) phenyl]isoxazole-3-carboxamide) is a highly potent, isoxazole-based, non-geldanamycin HSP90 inhibitor that inhibits the ATPase activity of HSP90, and leads to misfolding of client proteins [9, 10]. AUY922 has significant antitumor activity in a wide range of cancer cell lines and inhibits tumor growth in murine xenograft models [9–13]. In a preceding global Phase I study in Western patients (CAUY922A2101), the recommended Phase II dose (RP2D) of AUY922 was determined to be 70 mg/m^2^ intravenous (IV) once-weekly [14]. Phase II studies have been initiated in patients with HER2-positive breast cancer, gastric cancer and non-small cell lung cancer to further investigate the safety profile and clinical efficacy of AUY922 as a single agent and in combination with other agents. In these global Phase II studies, AUY922 was well tolerated with promising clinical activity as single-agent therapy, as well as in combination with other agents, in some sub-populations with actively progressing disease [15, 16].

In the present open-label, Phase I dose-escalation study, the safety, pharmacokinetics (PK) and clinical efficacy of AUY922 were evaluated in Japanese patients with advanced solid tumors. The primary objective was to determine the maximum tolerated dose (MTD) of AUY922 as a single agent when administered intravenously on a once-weekly schedule. Secondary objectives were to characterize the safety and tolerability of AUY922 treatment, evaluate the preliminary antitumor activity of AUY922 as a single agent and observe the PK profile of AUY922 and its metabolite.

## Materials and methods

### Patient population

Adult patients (aged ≥20 years) with histologically confirmed, advanced solid tumors whose disease had progressed on at least one line of standard systemic therapy, or for whom no standard therapy existed, were eligible. Inclusion criteria included Eastern Cooperative Oncology Group performance status ≤2 and life expectancy ≥12 weeks. Laboratory parameters required were absolute neutrophil count ≥1.5 × 10^9^/l, hemoglobin ≥8.5 g/dl, platelets ≥100 × 10^9^/l, potassium, calcium, magnesium, phosphorus within normal limits or correctable with supplements, aspartate aminotransferase and alanine aminotransferase ≤2.5 × upper limit of normal (ULN), serum bilirubin ≤1.5 × ULN, serum albumin >2.5 g/dl, and serum creatinine ≤1.5 × ULN or 24-hour clearance ≥50 ml/min.

Exclusion criteria included central nervous system metastases, acute or chronic liver or renal disease and previous treatment with histone deacetylase or HSP90 inhibitors. Patients were excluded who had clinically significant heart disease, QTc ≥450 ms on screening electrocardiogram (ECG), ischemic heart disease, heart failure, ECG abnormalities, atrial fibrillation, atrial flutter or ventricular arrhythmias including ventricular tachycardia or Torsades de Pointes, or a history (or family history) of long QT syndrome. Patients receiving any medication that had a risk of prolonging the QTcF interval or inducing Torsades de Pointes, and those with disorders known to be caused by a deficiency in bilirubin glucuronidation (e.g., Gilbert’s syndrome), were also excluded.

This trial was conducted in accordance with the Declaration of Helsinki and the Good Clinical Practice guidelines (Japanese Ministry of Health, Labour and Welfare). All studies were performed after approval by local ethical committee/institutional review board. Written informed consent was obtained from all patients before screening.

### Dosing and administration

AUY922 was administered by IV infusion over 1 h, once every week (Days 1, 8, 15 and 22) until disease progression, unacceptable toxicity, or withdrawal by investigator decision or patient refusal. The starting dose was 8 mg/m^2^, and treatment cycles were 28 days. Intra-patient dose escalation was not permitted. An adaptive Bayesian logistic regression model (BLRM), guided by the escalation with overdose control (EWOC) principle, was used to guide dose escalations [17]. The EWOC principle mandates the potential doses recommended for the next cohort and the estimated MTD have less than 25 % posterior probability of DLT in the excessive toxicity interval (33, 100 %). The information about dose-limiting toxicity (DLT) available from the CAUY922A2101 study at the time of the start of this study supported a starting dose of 8 mg/m^2 [^50 % of the highest dose (16 mg/m^2^) associated with no DLTs]. Toxicities at both the 8 and 16 mg/m^2^ dose levels in that study had been mild, and the preliminary PK results had shown no accumulation of the drug up to a dose of 16 mg/m^2^. The occurrence of DLTs was evaluated during Cycle 1. A minimum of three patients were enrolled in a cohort, and the estimated MTD was continuously updated using the BLRM, and a minimum of six patients were planned to be enrolled at the MTD level. The final recommended dose was based on overall safety assessments and MTD estimated by the BLRM, which was the dose of AUY922 with the highest posterior probability of DLT in the target interval (16, 33 %) among the doses fulfilling the EWOC principle [18]. A minimum of 15 patients were required for the BLRM model to determine the MTD. AUY922 was supplied as 10-ml ampoules of a 5-mg/ml solution, which was diluted into 5 % dextrose or glucose to a maximum infusion volume of 500 ml, under aseptic conditions and protected from light to prevent the photolabile drug from decomposition. Doses were individually adjusted according to body surface area measured at baseline.

### Safety assessments

Toxicity was graded according to the National Cancer Institute Common Toxicity Criteria version 3.0. DLTs were defined as clinically relevant adverse events (AEs; mainly Grade 3 or 4) or abnormal laboratory values, occurring within 28 days following the first dose of AUY922 in Cycle 1. Tumor response was assessed by computed tomography or magnetic resonance imaging, and using Response Evaluation Criteria in Solid Tumors version 1.0 for efficacy evaluations.

Based on reports of Grade 1–3 visual symptoms after weekly administration of AUY922 at dose levels of ≥40 mg/m^2^ in the CAUY922A2101 study, standard ophthalmological assessments were implemented at baseline, at the time of reported visual symptom(s) (if any) and at the end of treatment.

### Pharmacokinetic analysis

Validated liquid chromatography-tandem mass spectrometry assay was used for PK assessments of AUY922 and its glucuronide metabolite BJP762. PK assessments were carried out on blood samples obtained pre-infusion and at 5, 15, and 30 min and 1 h during infusion, followed by 5 and 30 min and 1, 2, 4, 5, 8, 24, 48 and 72-h post-infusion on Cycle 1 Day 1 and Cycle 2 Day 1. A non-compartmental analytical method was used to calculate PK parameters of maximum observed concentration (*C*_max_), time at which *C*_max_ occurred (*T*_max_), terminal half-life (T_1/2_), and area under the curve (AUC), for AUY922 and BJP762 in blood, utilizing WinNonlin Pro version 5.2.

## Results

### Patient characteristics and treatment

A total of 31 patients were treated in seven dose cohorts (8, 16, 22, 28, 40, 54 and 70 mg/m^2^) between November 2008 and July 2011 (Table 1). Median duration of drug exposure was 7.3 weeks (range 0.1–58.1 weeks) and 55 % of patients underwent 1 or 2 treatment cycles [7 patients (23 %) and 10 patients (32 %), respectively]. The median relative dose intensity was 1.0 (range 0.7–1.0). At the time of data cut-off (5 July 2011), two patients were still receiving treatment on the study. The remaining 29 patients discontinued the study treatment, mainly due to disease progression (27 patients), and two patients discontinued as a result of AEs related to study drug (one patient each in the 54 and 70-mg/m^2^ cohorts).

**Table 1:** Patient demographics, and baseline disease characteristics

Characteristic	AUY922 dose (mg/m^2^)							
	8 (*n* = 3)	16 (*n* = 3)	22 (*n* = 3)	28 (*n* = 5)	40 (*n* = 3)	54 (*n* = 6)	70 (*n* = 8)	Total, *n* (%) (*N* = 31)
Mean age, years	51.3	61.7	52.7	53.6	62.0	62.3	59.4	58.1
Gender, *n*								
Male	2	1	3	2	1	2	4	15 (48)
ECOG PS, *n*								
0	2	3	2	3	3	4	4	21 (68)
1	1	0	1	2	0	2	4	10 (32)
Stage (current), *n*								
IV	3	3	3	3	3	6	8	29 (94)
IVa	0	0	0	1	0	0	0	1 (3)
IVb	0	0	0	1	0	0	0	1 (3)
Tumor type, *n*								
Rectum	0	10	2	0	0	2	5	10 (32)
Colon	1	1	1	0	1	2	1	7 (23)
Breast	0	0	0	2	1	1	1	5 (16)
Head and neck	1	0	0	0	1	0	0	2 (7)
Pancreas	0	0	0	1	0	0	1	2 (7)
Stomach	1	1	0	0	0	0	0	2 (7)
Eesophagus	0	0	0	1	0	0	0	1 (3)
Gall bladder ducts	0	0	0	1	0	0	0	1 (3)
Other	0	0	0	0	0	1	0	1 (3)

*ECOG PS* Eastern Cooperative Oncology Group performance status

### Safety and tolerability

The most common AEs, regardless of relationship to study drug, were diarrhea (65 %), night blindness (42 %), nausea and fatigue (both 29 %). Mild-to-moderate diarrhea (65 %), night blindness (42 %) and nausea (23 %) were the most commonly reported AEs possibly related to AUY922 treatment across all doses (Table 2). Visual toxicities, including night blindness, photopsia, cataract, eye disorder, optic neuritis and blurred vision were observed at dose levels of 22–70 mg/m^2^; all were Grade 1 or 2. No patients discontinued AUY922 treatment due to the visual toxicities, which were reversible upon discontinuation of treatment. None of the visual AEs were reported as DLTs. Fifteen patients (48 %) experienced AEs requiring dose modification or interruption. Of these, night blindness (six patients) and eye disorder (two patients) were reported. Eight patients (26 %) experienced serious AEs (SAEs) and SAEs considered to be related to the study drug were reported in two patients. One patient died during the study as a result of disease progression, which was considered to be unrelated to study drug.

**Table 2:** Most common adverse events (≥10 % and Grade 3/4) potentially related to AUY922 treatment

Adverse event, *n* ^a^	Grade	AUY922 dose (mg/m^2^)							
		8 (*n* = 3)	16 (*n* = 3)	22 (*n* = 3)	28 (*n* = 5)	40 (*n* = 3)	54 (*n* = 6)	70 (*n* = 8)	Total, *n* (%) (*N* = 31)
Diarrhea	All	0	0	2	4	2	5	7	20 (65)
	3/4	0	0	0	0	0	1	0	1 (3)
Night blindness	All	0	0	2	2	1	5	3	13 (42)
	3/4	0	0	0	0	0	0	0	0
Nausea	All	0	0	0	0	2	2	3	7 (23)
	3/4	0	0	0	0	0	0	0	0
Decreased appetite	All	0	1	1	0	0	1	3	6 (19)
	3/4	0	0	0	0	0	1	0	1 (3)
Fatigue	All	0	0	0	2	0	3	1	6 (19)
	3/4	0	0	0	0	0	1	0	1 (3)
Rash	All	0	2	0	1	0	2	1	6 (19)
	3/4	0	0	0	0	0	0	0	0
Vomiting	All	0	0	0	0	1	1	3	5 (16)
	3/4	0	0	0	0	0	0	0	0
Headache	All	0	0	0	0	0	0	3	3 (10)
	3/4	0	0	0	0	0	0	0	0
Lymphopenia	All	0	0	0	2	0	1	0	3 (10)
	3/4	0	0	0	0	0	0	0	0
Photopsia	All	0	0	1	1	0	0	1	3 (10)
	3/4	0	0	0	0	0	0	0	0
Pruritis	All	0	1	0	0	0	1	1	3 (10)
	3/4	0	0	0	0	0	0	0	0

Patients who experienced more than one occurrence of the same event are only counted once within each category

^a^By preferred term

The dose-determining set (DDS) consisted of (1) all patients who received at least three doses of AUY922 within the first cycle, were observed for ≥28 days following the first dose, and completed all safety evaluations in Cycle 1, or (2) any patient who had a DLT within Cycle 1. The DDS was used for the BLRM analyses in the determination of MTD. Among these patients (*n* = 28), one patient (3.6 %) in the 54-mg/m^2^ cohort experienced DLTs; two AEs (Grade 3 fatigue and Grade 3 decreased appetite) were considered to be DLTs and both resolved to Grade 1 within 8 days. Two patients who received the 70 mg/m^2^ dose required repeated dose interruption due to visual adverse events, and both patients ultimately received a dose reduction to 54 mg/m^2^. Although the BLRM would have permitted dose escalation beyond 70 mg/m^2^, a decision to stop further dose escalation was taken based on an assessment by investigators of the potential risk of visual toxicities. Visual toxicities at the higher doses (22 mg/m^2^ and above) among those tested were observed, most commonly night blindness and photopsia, although these were only Grade 1 or 2. As a result, the MTD was not determined, and the RP2D was, therefore, declared as 70 mg/m^2^ once-weekly IV over 1 h.

### Pharmacokinetics

**Fig. 1:**
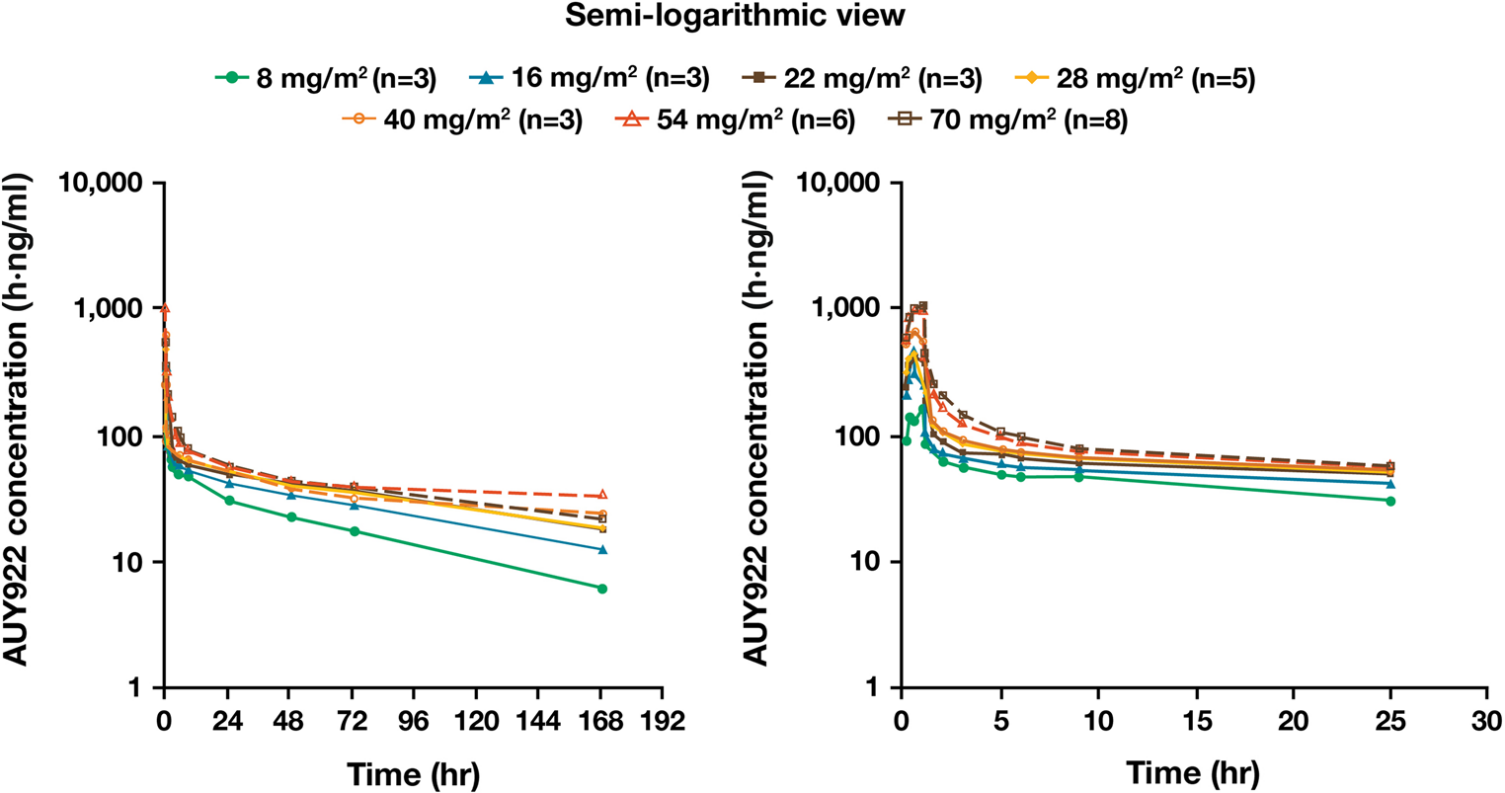
Mean AUY922 concentration–time profiles in blood on Cycle 1 Day 1

**Fig. 2:**
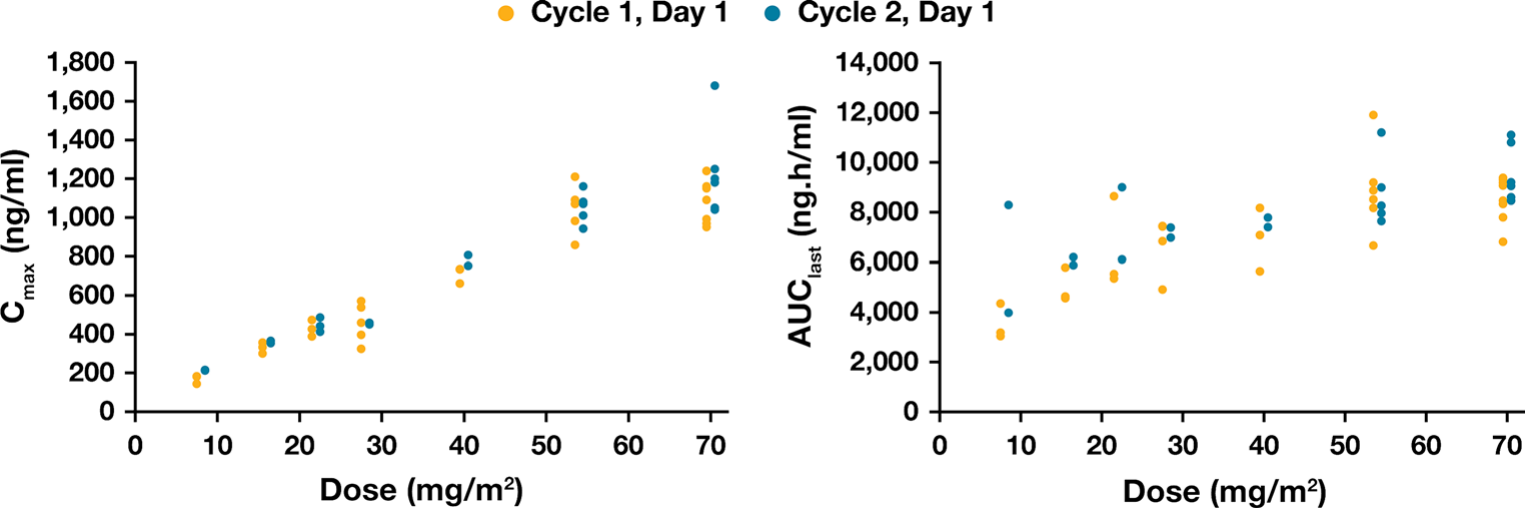
Relationship between AUY922 dose and blood pharmacokinetics parameters

AUY922 reached peak concentrations in blood around the end of the infusion. Following the initial rapid decline in concentration levels after the IV administration was completed, blood AUY922 concentration declined slowly, with a mean T_1/2_ of 127 h at 70 mg/m^2^ (Table 3; Fig. 1). The T_1/2_ was prolonged with increasing dose (64 h at 8 mg/m^2^ to 127 h at 70 mg/m^2^). *C*_max_ for both AUY922 and the metabolite BJP762 generally increased in a dose-proportional manner over the entire dose range. AUC_last_ of AUY922 increased with dose (from 8 to 28 mg/m^2^), but was saturated at higher dose levels (Fig. 2; Tables 3, 4). Due to limited sampling time points, the plasma concentration–time profile could not be fully characterized; *C*_max_ and AUC for AUY922 in plasma had a tendency to increase in a dose-proportional manner even at the higher dose levels (40–70 mg/m^2^). Blood PK profiles for AUY922 on Day 1 of Cycle 2 were similar to those on Day 1 of Cycle 1. The geometric mean of accumulation ratios for *C*_max_ (Day 1 of Cycle 1 to Day 1 of Cycle 2) ranged from 1.01 to 1.28. The ratios for AUC_last_ (Day 1 of Cycle 1 to Day 1 of Cycle 2) ranged between 0.992 and 1.60. Hence, there was no significant drug accumulation with once-weekly IV doses of AUY922.

**Table 3:** Summary of PK parameters (mean ± SD, unless otherwise stated) at Cycle 1 Day 1 for blood AUY922 [28–70 mg/m^2^ (four highest doses)]

AUY922 PK parameter	AUY922 dose (mg/m^2^)			
	28 (*n* = 5)	40 (*n* = 3)	54 (*n* = 6)	70 (*n* = 8)
*T*_max_ [median, h (range)]	0.50 (0.48–1.07)	1.05 (0.50–1.05)	0.76 (0.48–1.17)	1.02 (0.23–1.17)
*C*_max_ (ng/ml)	457 ± 101	710 ± 42	1,050 ± 118	1,100 ± 118
AUC_(0–last)_ (h·ng/ml)	6,810 ± 1,090	6,960 ± 1,270	8,880 ± 1,710	8,540 ± 895
AUC_(0–inf)_ (h·ng/ml)	9,550 ± 2,460	11,400 ± 3470^a^	12,300 ± 2720^a^	12,600 ± 1,720
CL (l/h)	4.79 ± 1.68	5.74 ± 1.16^a^	7.24 ± 1.97^a^	8.60 ± 1.30
V_z_ (l)	646 ± 111	980 ± 132^a^	1,190 ± 151^a^	1,570 ± 293
T_1/2_ (h)	98.7 ± 23.0	123.0 ± 40.8^a^	120.0 ± 28.5^a^	127.0 ± 18.8

*PK* pharmacokinetics, *SD* standard deviation

^a^Data missing for one patient

**Table 4:** Summary of PK parameters (mean ± SD, unless otherwise stated) at Cycle 1 Day 1 for blood BJP762 [28–70 mg/m^2^ (four highest doses of AUY922)]

BJP762 PK parameter	AUY922 dose (mg/m^2^)			
	28 (*n* = 5)	40 (*n* = 3)	54 (*n* = 6)	70 (*n* = 8)
*T*_max_ [median, h (range)]	1.07 (1.05–1.17)	1.05 (1.05–1.07)	1.08 (1.02–1.22)	1.13 (1.00–1.23)
*C*_max_ (ng/ml)	611 ± 201	964 ± 775	1,060 ± 569	1,330 ± 904
AUC_(0–last)_ (h·ng/ml)	3,700 ± 2,170	5,690 ± 5,250	6,320 ± 4,650	5,530 ± 3,320
AUC_(0–inf)_ (h·ng/ml)	3,940 ± 2,320	5,830 ± 5,300	6,770 ± 5200^a^	5,020 ± 3340^b^
T_1/2_ (h)	59.1 ± 28.8	33.1 ± 12.1	49.1 ± 24.0^a^	46.5 ± 27.9^b^

*PK* pharmacokinetics, *SD* standard deviation

^a^Data missing for one patient

^b^Data missing for two patients

### Efficacy

**Fig. 3:**
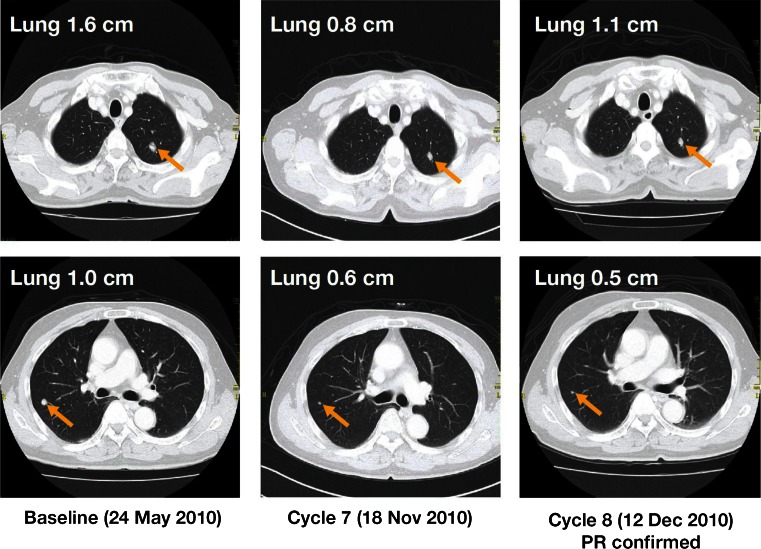
Computed tomography scans of lung metastases in a patient with a confirmed PR following treatment with AUY922 54 mg/m^2^ (63 years old, male, primary rectal carcinoid tumor)

One patient (rectal carcinoid tumor with lung metastatic lesions) achieved a confirmed partial response (PR) for a duration of >7 months (Table 5; Fig. 3). Ten patients (32 %) achieved a best overall response of stable disease (SD) lasting ≥8 weeks, including five out of the eight patients (63 %) in the 70-mg/m^2^ cohort; no patients achieved a complete response. The disease control rate (DCR; PR + SD) across all dose levels was 36 % (Table 5).

**Table 5:** Best overall response (Response Evaluation Criteria in Solid Tumors)

Response, *n*	AUY922 dose (mg/m^2^)							
	8 (*n* = 3)	16 (*n* = 3)	22 (*n* = 3)	28 (*n* = 5)	40 (*n* = 3)	54 (*n* = 6)	70 (*n* = 8)	Total, *n* (%) (*N* = 31)
Complete response	0	0	0	0	0	0	0	0
Partial response	0	0	0	0	0	1	0	1 (3)
Stable disease	1	1	0	1	1	1	5	10 (32)
Progressive disease	2	2	3	4	2	3	3	19 (61)
Unknown	0	0	0	0	0	1	0	1 (3)
Overall response rate (CR + PR)	0	0	0	0	0	1	0	1 (3)
Disease control rate (CR + PR + SD)	1	1	0	1	1	2	5	11 (36)

## Discussion

There are a number of HSP90 inhibitors under clinical development, both as single agents and in combination with other agents [19, 20]. Hepatotoxicity has been reported in both the early and late stages of development of geldanamycin-based HSP90 inhibitors [21, 22]. In this study in Japanese patients with advanced solid tumors, single-agent AUY922 demonstrated an acceptable safety profile at dose levels of 8–70 mg/m^2^ with potential clinical activity (DCR 36 %). The MTD was not established, and although the BLRM would have permitted further dose escalation, a decision was made not to escalate the dose any further than the well-tolerated dose of 70 mg/m^2^ based on the potential risk of visual toxicity, the symptoms of which were similar to those reported in the preceding global phase I study (CAUY922A2101), and the RP2D was thus declared as 70 mg/m^2^once-weekly.

Hepatotoxicity was not reported as a frequent AE suspected to be related to study drug, or as a DLT; the most common AEs suspected to be related to this study drug included Grade 1 or 2 diarrhea (65 %), night blindness (42 %) and nausea (23 %). Only Grade 1 or 2 visual AEs (most commonly night blindness and photopsia) were reported at the 22–70 mg/m^2^ dose levels. Similar safety findings were observed in the preceding global Phase I CAUY922A2101 study [14]. Visual disturbances have been reported with other geldanamycin and non-geldanamycin HSP90 inhibitors [23–25]. These visual AEs are regarded as class adverse effects, which are possibly related to tissue distribution of water-soluble agents facilitating a high retina:plasma concentration ratio, as well as the retinal elimination profile [26]. The safety profile of AUY922 was similar to that reported in the preceding CAUY922A2101 study [14], and ongoing Phase II studies [15, 16]. *C*_max_ for AUY922 in blood increased generally in a dose-proportional manner over the entire dose range. Blood PK parameters of AUY922 in Japanese patients were also comparable to those observed in non-Japanese patients in the CAUY922A2101 study [14]. AUC for AUY922 in blood increased with dose at lower doses, but was saturated at higher doses. This less than dose-proportional increase in blood AUY922 is likely caused by a concentration-dependent and saturable blood cell partition of AUY922. There was no significant drug accumulation following once-weekly intravenous infusion of AUY922.

In summary, AUY922 has shown an acceptable safety profile and demonstrated promising clinical activity in Japanese patients, with one patient achieving a confirmed prolonged PR, and several patients achieving long duration SD at higher dose levels.
